# THE GOOD OLD DAYS?: LATER-BORN COHORTS REPORT DIFFERENT STRESSORS IN DAILY LIFE COMPARED TO EARLIER-BORN COHORTS

**DOI:** 10.1093/geroni/igad104.0923

**Published:** 2023-12-21

**Authors:** Rachel Koffer

**Affiliations:** Arizona State University, Phoenix, Arizona, United States

## Abstract

In addition to the total amount of exposure to stressors in daily life, the types of stressors experienced (e.g., work stress, arguments), and the relative spread of the stressor types experienced (stressor diversity) is key to emotional and physical well-being across adulthood and aging. While daily stress processes and their links to health and well-being have been studied extensively, historical changes in daily stress processes have been largely unexplored. To examine secular trends in the types of daily stressors experienced across adulthood, we use data from two independent cohorts of the National Study of Daily Experiences surveyed 18 years apart (1995/1996 cohort: n = 1,499 adults aged 20 to 74 years; 2013/2014 cohort: n = 782 adults aged 25 to 75, stratified by age and gender to match the 1995-96 cohort). General linear models are used to test cohort differences in total stressor exposure, exposure to specific types of stressors, and stressor diversity, all controlling for age, income relative to cohort, education relative to cohort, gender, and chronic conditions. While the 2013/2014 cohort experience higher exposure to stressors in daily life than the 1995/1996 cohort, the cohorts experience the same stressor diversity, which decreases with older age. The specific stressor types experienced differ by cohort: adults in 2013/2014 report more arguments, avoided arguments, and work stressors, but less discrimination stressors and “other” stressors than in 1995/1996. These results highlight the sociohistorical embedding of daily stressor experiences. Sociodemographic differences and implications for future research and clinical practice are discussed.

